# Growth Trajectories of Health Behaviors from Adolescence through Young Adulthood

**DOI:** 10.3390/ijerph121113711

**Published:** 2015-10-28

**Authors:** Nora Wiium, Kyrre Breivik, Bente Wold

**Affiliations:** 1 Department of Psychosocial Science, Faculty of Psychology, University of Bergen, Christiesgate 12, Bergen N-5020, Norway; 2 Uni Research, Regional Centre for Child and Youth Mental Health and Child Welfare, Krinkelkroken 1, Bergen N-5014, Norway; E-Mail: Kyrre.Breivik@uni.no; 3 Department of Health Promotion and Development, Faculty of Psychology, University of Bergen, Christiesgate 13, Bergen N-5020, Norway; E-Mail: Bente.Wold@uib.no

**Keywords:** drunkenness, fruit intake, latent class growth analysis, physical activity, smoking

## Abstract

Based on nine waves of data collected during a period of 17 years (1990–2007), the present study explored different developmental trajectories of the following unhealthy behaviors: regular smoking, lack of regular exercise, lack of daily fruit intake, and drunkenness. A baseline sample of 1195 13-year-old pupils was from 22 randomly selected schools in the Hordaland County in western Norway. Latent class growth analysis revealed three developmental trajectories. The first trajectory was a conventional trajectory, comprising 36.3% of participants, who showed changes in smoking, physical exercise, fruit intake, and drunkenness consistent with the prevailing age specific norms of these behaviors in the Norwegian society at the time. The second trajectory was a passive trajectory, comprising 25.5% of participants, who reported low levels of both healthy and unhealthy behaviors during the 17-year period. The third trajectory was an unhealthy trajectory, comprising 38.2% of participants, who had high levels of unhealthy behaviors over time. Several covariates were examined, but only sex and mother’s and father’s educational levels were found to be significantly associated with the identified trajectories. While these findings need to be replicated in future studies, the identification of the different trajectories suggests the need to tailor intervention according to specific needs.

## 1. Introduction

Chronic diseases such as cancer, heart disease, and diabetes are major causes of disability, and account for about 60% of all deaths worldwide [1]. These chronic diseases have been linked to a small number of preventable unhealthy behaviors such as alcohol and drug misuse, tobacco use, physical inactivity, and unhealthy diet [1,2]. Over the years, several regulations and recommendations (e.g., the World Health Organization (WHO) recommendation of five servings of fruits and vegetables per day and physical activity, 30 to 60 min per day) have been implemented in different countries to promote healthy lifestyles among young and old alike. However, rates of several unhealthy behaviors in the general population are still high enough to raise public health concerns in many countries. Among young people, high levels of unhealthy behaviors such as smoking and alcohol consumption are already evident at the early stages of adolescence, and tend to persist throughout the entire period [3,4]. The World Health Organization has defined adolescents, young people and adults as individuals in the age group of 10–19, individuals in the age group of 10–24, and individuals in the age group of 20–64, respectively [5,6]. We apply these definitions in the present study. 

The period between adolescence and young adulthood has also been seen as a crucial phase where unhealthy behaviors can develop as a result of changing roles, responsibilities, and situations. Unhealthy behaviors do not only persist throughout adolescence but may also continue into adulthood [7,8]; individuals who smoke during adolescence have also been found to smoke during adulthood. Stability in both healthy and unhealthy behaviors such as physical activity, alcohol consumption, fruit and vegetable consumption has also been observed between adolescence and adulthood [7,8,9]. 

The stability in health behaviors over time is suggested to reflect lifestyles that are influenced by both life choices (a process of agency, where course of action is evaluated and chosen) and life chances (e.g., *class circumstances* and to some extent age, gender, and living conditions) [10]. Cockerham’s health lifestyle theory describes how the interaction between chances and choices may lead to different actions and practices. Accordingly, that high socioeconomic status (SES) individuals are more likely to engage in healthy behaviors than low-SES individuals, may not only reflect choices but also class position which may “…either empower or constrain choices as choices and chances work off each other to determine behavioral outcomes” (Cockerham [10], page 55). The behavior is further likely to be sustained through the process of modelling or the feedback that is obtained. 

Health behaviors have been found to co-occur; de Vries *et al.* [11] found several clusters among five health behaviors (physical activity, alcohol consumption, non-smoking, fruit and vegetable consumption) in an adult population; independent clustering of unhealthy and healthy behaviors were replicated in low, moderate, and high educational subgroups. Berrigan *et al.* [12] also found a clustering of unhealthy and healthy behaviors where most US adults adhered to the recommendation for smoking and alcohol but not to the recommendation for exercise, dietary fat, fruit and vegetable consumption. 

The co-occurrence of unhealthy behaviors is particularly evident during adolescence. For example, adolescents who smoke regularly have also been found to engage in high alcohol consumption [13] and risky sexual behavior [14]. Associations have also been found between high alcohol consumption and risky sexual behaviors [15]. Furthermore, associations have been found between several unhealthy behaviors and lower levels of healthy behaviors such as physical activity [16] and fruit and vegetable intake [17]. 

The high prevalence of risky or unhealthy behaviors during adolescence has been linked to several reasons, among them, what Steinberg [18] has referred to as the brain’s “socio-emotional system” that can lead to increased reward-seeking among adolescents, especially when they are in the presence of their peers. Steinberg [18] explains that “…the pubertal transition is associated with a substantial increase in sensation-seeking that is likely due to changes in reward salience and reward sensitivity resulting from a biologically-driven remodeling of dopaminergic pathways…” (page 92). It is these pathways that have been referred to as the social-emotional system and the system’s effect may vary from person to person. This explanation may hold for experimentation with and use of alcohol and tobacco during adolescence. For factors that are related to the declines in physical activity and intake of fruit and vegetables observed during adolescence, earlier studies have produced mixed findings. However, there have been calls to look into developmental and environmental factors [19,20]. 

The transition into adulthood has been associated with an increased or a decreased engagement in certain health behaviors because of the different expectations, social roles, and responsibilities (e.g., parenthood, marriage, increases in postsecondary education and employment) that may accompany the transition. In line with Cockerham’s [10] healthy lifestyle theory, age and gender are examples of life chances that may somewhat influence the progress of behavior; accordingly women, and people, as they get older, tend to make healthier choices to some extent. Nelson *et al.* [21] found a decline in physical activity and diet quality (e.g. fruits, vegetables and milk) and an increase in sedentary behaviors and substance use among emerging adults in academic institutions. Bingham *et al.* [22] found an increase in at-risk drinking from adolescence to young adulthood among never-married young adults, especially among men. Little *et al.* [23] however, observed a decline in the rate of alcohol consumption for individuals becoming parents during emerging adulthood. Becoming a parent has also been associated with a decrease in physical activity [24]. Smoking cessation has been observed among pregnant women, emerging adults, and young mothers [25,26]. A review of the literature suggests that the patterns described above are increasingly common during the transition to adulthood. Demographic factors such as parents’ educational background in addition to age and sex may play a moderating or mediating role. 

From the literature reviewed in the preceding paragraphs, the design of previous studies assessing clustering of multiple health behaviors appears to be mostly cross-sectional or short-term longitudinal, limiting the extent to which different developmental trajectories can be assessed over a longer period of time. With a longitudinal data set spanning over a period of 17 years (from ages 13 to 30), the present study explores different developmental trajectories regarding smoking, physical activity, fruit intake, and drunkenness. To proceed with this, latent class growth analysis (LCGA) is applied. LCGA entertains the possibility that a given sample population may consist of a number of “latent” subpopulations or classes with qualitatively different development patterns on the outcome variable(s) [27]. While heterogeneity between subpopulations is assumed, homogeneity is assumed within a subpopulation. This notion of distinct developmental patterns for different subpopulations reflects the theoretical literature in fields such as psychology about the heterogeneity of growth trajectories that may exist within a larger population in areas like personality, language, prosocial and antisocial development [27,28]. Depending on their characteristics, different groups of individuals may follow distinct developmental trajectories. For example, Moffitt [29] in a study observed that childhood-onset and adolescent-onset antisocial behaviors were distinct trajectories that reflected the timing and extent of antisocial behaviors that individuals in these distinct trajectories were involved in. LCGA has been used to study developmental patterns of health behaviors such as alcohol use [30] and physical activity [31]. 

Based on the above theoretical and empirical discussion, and in view of the four health behaviors that are simultaneously examined, we expect to observe several subpopulations with different trajectories including: (1) a latent class which is involved in low levels of unhealthy behaviors across the 17-year period. We expect that this subpopulation would consist of rather many participants who have parents with high educational background; (2) another latent class that is defined by increasing drunkenness over time. It is hypothesized that this subpopulation will comprise of individuals who are pursuing education during young adulthood as well as not having a cohabiter or children; and (3) a latent class which is characterized by both healthy and unhealthy behaviors during the transition to adulthood. We hypothesize that this subpopulation would consist of quite a few who are married/cohabiting and/or have children. We examine also the influence of sex on the different growth trajectories. The identification of different subpopulations and their associated factors will likely assist in designing tailored intervention programmes that will meet the specific needs of the subgroups.

## 2. Methods

The present study used data from the Norwegian Longitudinal Health Behavior Study, which investigated among others several indicators of health behaviors of participants over a 17-year period. 

### 2.1. Baseline Sample

The baseline sample was obtained from a list of schools of 13-year-old pupils in Norway, which was provided by the Ministry of Education. Twenty-two schools comprising 54 school classes were randomly selected by picking out every 5th school from an alphabetical list of schools in Hordaland County in western Norway. In autumn 1990, 1195 13-year olds in the seventh grade from the schools that were selected were invited to participate in the longitudinal study. All pupils in the chosen school classes were selected for the study sample. Norwegian school classes are typically homogeneous in age. 927 pupils accepted the invitation and participated in the study. Of the 268 non-participants, 222 were because parents did not return written consent for their children to participate and 46 because pupils did not want to participate. Three pupils who participated in the study had incomplete responses on most of the variables and were therefore excluded from the analysis. The sample size for 1990 that was included in the data analysis was therefore 924 pupils, that is, 77% of the baseline sample; 55% were males. During the first two years of data collection, new students who got enrolled in the participating schools were invited to participate in the study. Thus, the number of participants increased to 1134 during these years. A subsample of 1057 individuals (who had some responses to the items being focused on in the present study) was used in the analyses.

### 2.2. Data Collection

Data were collected nine times from participants from age 13 in 1990 until age 30 in 2007 (see Table 1). For the years 1990–1992, data were collected from pupils during an ordinary class hour, which usually last for 45 min. The project coordinators from the Research Centre of Health Promotion, University of Bergen, Norway, were responsible for the administration of the data collection. From 1993, participants were contacted through the post and were sent a prepaid envelope to return their completed questionnaires. In 2007, 120 participants chose to complete their questionnaire online, rather than to return it through the post. Students were assured confidentiality during all waves of data collection and the study was approved by the Norwegian Data Inspectorate. 

**Table 1:** Number of participants by age and year of data collection.

Year	1990	1991	1992	1993	1995	1996	1998	2000	2007
Age	13	14	15	16	18	19	21	23	30
N	924	958	936	789	779	643	634	630	535

### 2.3. Variables

The items used to measure the study variables: smoking, physical activity, fruit intake, and drunkenness were adopted from the Health Behavior in School-aged Children (HBSC) study which is a WHO cross-national survey. These items have been used in the WHO cross-national surveys since 1983 [32] and were included in all the nine data collection years of the Norwegian Longitudinal Health Behavior Study. 

*Regular smoking* was measured as follows: “Have you ever smoked tobacco?” Responses were coded (0) no and (1) yes. Participants answering “yes” were asked a second question: “How often do you smoke?” The response was coded using a four-point scale: (1) “Not at all”; (2) “Less than once a week”; (3) “Every week”; and (4) “Every day”. Smoking status was computed using the two items; options (1) and (2) on the second item and (0) on the first item meant “non-smoking or occasional smoking” while (3) and (4) on the second item meant “regular smoking”.

*Lack of regular exercise* was measured with the following global measure: “In your leisure time, how often do you do sports or exercise until you are out of breath or sweat?” The response was coded using a seven-point scale: (0) never; (1) less than once per month; (3) 1–3 times per month; (4) once per week; (5) 2–3 times per week; (6) 4–6 times per week; and (7) every day. The response categories were recoded into two: “physical activity once per week or less” (*i.e.*, lack of regular exercise) and “physical activity two or more times per week”. A one-week test-retest reliability of the item among eighty 14-year-old participants was relatively high (*r* = 0.78). 

*Lack of daily fruit intake.* Participants were asked to indicate the frequency of their fruit intake in the past three months. Responses were (1) seldom or never; (2) 1–2 times a week; (3) 3–6 times a week; (4) once a day; and (5) several times a day. The response categories were recoded into two: “fruit intake six or fewer times a week” (*i.e.*, lack of daily fruit intake) and “fruit intake once or several times a day”. A one-week test-retest reliability of the item among eighty 14-year-old participants was (*r* = 0.70).

*Drunkenness.* Participants were asked to indicate the number of times they have been aware of being drunk in the past 6 months. Responses were (1) never in the past six months; (2) once; (3) 2–4 times; (4) 5–10 times; (5) 11–24 times; (6) 25–49 times; and (7) At least 50 times. The response categories were recoded into two categories: “four or fewer times drunk in the past six months” and “at least five times drunk in the past six months” (*i.e.*, drunkenness).

*Covariates measured at baseline:* sex of participants (*i.e.*, whether a participant was a male or female).

*Covariates measured in 1996:* mother and father’s self-report of educational level: (responses ranged from [1] primary school completed to [5] more than four years of University/college education). 

*Covariates measured in 2007:* participants were asked to indicate whether or not they had children, their civil status (whether or not they were married or cohabiting) and their occupational status (several options were offered among them whether participants were students).

### 2.4. Data Analysis

Frequency distribution was run for each of the four health behaviors across the 17-year period. As previously mentioned, the development of the various health behaviors over time was estimated by the use of LCGA, where the group status is not known but inferred from the data. Latent class growth analysis (LCGA) does not allow for variation within classes and assumes that the variance and covariance estimates of the growth factors are fixed to zero [27]. Because the LCGA analysis was exploratory, several trajectories, more than the three that were hypothesized were tested. Sex, educational level of father and mother, parenthood, civil status, and status as a student were included in model specification as covariates of class membership. The selection of the most optimal number of classes was based on a theoretical understanding of the trajectories as well as on statistical criteria such as the Akaike information criterion (AIC), the Bayesian Information Criterion (BIC) value [33], Lo *et al.* [34] Lo-Mendell-Rubin adjusted likelihood ratio test (LMR-LRT) statistic and the bootstrap likelihood ratio test (BLRT) statistic. As suggested by Nylund *et al.* [33] a non-significant p value from the LMR test was used as a stopping rule for the number of classes which should be derived. The LMR test might however overestimate the number of classes, hence this test was followed by inspection of other fit indices of which the BLRT and BIC have been found to perform best [33]. In general, smaller BIC as well as AIC values are preferable although the latter criterion is not always reliable. Classification accuracy of the individuals was assessed by entropy ranging between 0 and 1 where 1 is the best classification. The LCGA analyses were examined for non-convergence and local maxima and treated accordingly by increasing the number of random starts perturbations. 

All analyses were run using the Mplus statistical programme, version 7 [35], and the different models were estimated by the use of the full information maximum likelihood (FIML) estimator with robust standard errors (MLR) where the health behavior variables were treated as dichotomous (*i.e.*, categorized as 0 and 1, where the value 1 indicated an unhealthy behavior). Thus, regular smoking, lack of regular exercise, lack of daily fruit intake and drunkenness were referred to as unhealthy behaviors in the present study. The FIML method uses all available data and assumes that the data is at least Missing At Random (MAR). MAR exists when the probability of the missing data on a certain variable Y relies on other measured variables in the model, but not the values of variable Y itself [36], which is assumed to be the case in our data. This method is generally regarded as superior to standard ad hoc missing data routines methods such as listwise deletion and mean replacement [36,37].

## 3. Results 

### 3.1. Dropout Analysis

Drop-out analysis was undertaken by comparing baseline values of smoking, physical activity, fruit intake and drunkenness to examine whether those who dropped out of the study before age 30 differed from those who stayed on. Based on all the participants who participated in the study, the attrition rate was 49.4% ((1057 – 535) / 1057) at the age of 30. More males than females dropped out (63% *vs.* 37%; chi-square = 22.256, *p* < 0.001). For all the health behaviors at age 13, chi square analyses revealed no statistically significant difference between those who dropped out of the study before age 30 and those who stayed on until 30. 

### 3.2. Descriptive Analysis

Table 2 represents a frequency distribution of regular smoking, lack of regular exercise, lack of daily fruit intake and drunkenness for all nine time points (from age 13 to 30) for the overall sample. The proportion of individuals who exercised once a week or less increased throughout the entire period starting off at 21.2% at age 13 and reaching as much as 56.4% at age 30. For the remaining health behaviors, there was a curvilinear trend over time where the probability of engaging in unhealthy behaviors increased throughout adolescence, reached a peak at around age 21, and then declined. It is worth noting the great proportion of individuals who engaged in unhealthy behaviors especially in late adolescence/early young adulthood. At age 21, 42.8% smoked weekly or daily, 74.4% did not eat fruit every day, 62.7% were drunk five times or more in the past six months and 50.9% exercised once a week or less.

**Table 2:** Univariate proportions and counts for unhealthy behaviors over time (13–30 years).

Year (Age)	Unhealthy Behaviors				
	Regular Smoking (Weekly and Daily)	Lack of Regular Exercise (Physical Activity Once A Week or Less)	Lack of Daily Fruit Intake	Drunkenness (At Least 5 Times Drunk in the Past 6 Months)	*N* (range)
1990 (13)	4.8	21.2	43.3	1.6	699–912
1991 (14)	12.9	24.9	44.0	5.2	873–920
1992 (15)	23.0	28.7	51.4	17.2	877–908
1993 (16)	26.1	38.1	61.8	30.7	678–685
1995 (18)	37.0	48.6	69.1	55.0	736–749
1996 (19)	41.6	51.0	71.9	63.5	614–623
1998 (21)	42.8	50.9	74.4	62.7	566–570
2000 (23)	40.5	52.2	72.8	52.2	600–604
2007 (30)	25.0	56.4	55.0	36.2	514–515

Concerning descriptive analyses of the covariates, about 27% of 488 fathers and 18% of 548 mothers who responded to the item on education reported that they had more than 4 years of university or college education. In 2007 (*i.e.*, at the age of 30), 55% of 535 participants who responded to the item on children indicated that they were parents. About 74% of 535 participants reported that they were married or cohabiting, while about 8% of 528 participants who responded to the item on occupation indicated that they were students.

### 3.3. Latent Class Growth Analysis—Determining the Best Model

Table 3 presents findings from the LCGA that were undertaken with one to four-class solutions. We stopped increasing the number of classes at four due to the fact that the fit of the four-class model was not significantly better than the fit of the three-class model according to the LMR test (*p* = 0.099). This choice was supported by the fact that the four-class model did not add any distinctive features over the three-class model as two of its classes only differed quantitatively (on the level of health behaviors over time) rather than qualitatively (on the shapes of the growth curves). As the BIC value was smallest for the three-class model and the BLRT test revealed a significant difference between the two and three-class models, the latter was chosen as the most optimal. In addition, the three-class model was theoretically more meaningful than the two-class model. The number (and proportion) of participants belonging to the first, second, and third latent classes (or trajectories) were 384 (36.3%), 269 (25.4%), and 404 (38.2%), respectively, while the respective estimated probabilities of belonging to the trajectories were 86.7%, 89.0%, and 95.4%, with entropy of 0.782. 

**Table 3:** Fit indices for latent class growth analysis of unhealthy behaviors.

Number of Classes	Fit Indices					
	BIC	Adjusted BIC	AIC	Entropy	LMR-LRT *p*–Values	BLRT
1	29,600.631	29,562.517	29,541.073	–	–	–
2	27,251.877	27,172.473	27,127.797	0.811	0.0000	0.0000
3	26,603.467	26,482.773	26,414.866	0.782	0.0003	0.0000
4	26,198.357	26,036.373	25,945.235	0.790	0.0990	0.0000

**Notes:** BIC: Bayesian Information Criterion; AIC: Akaike information criterion; LMR–LRT: Lo-Mendell-Rubin adjusted likelihood ratio test; BLRT: Bootstrap likelihood ratio test.

### 3.4. Growth Trajectories and Associations with Covariates

**Figure 1:**
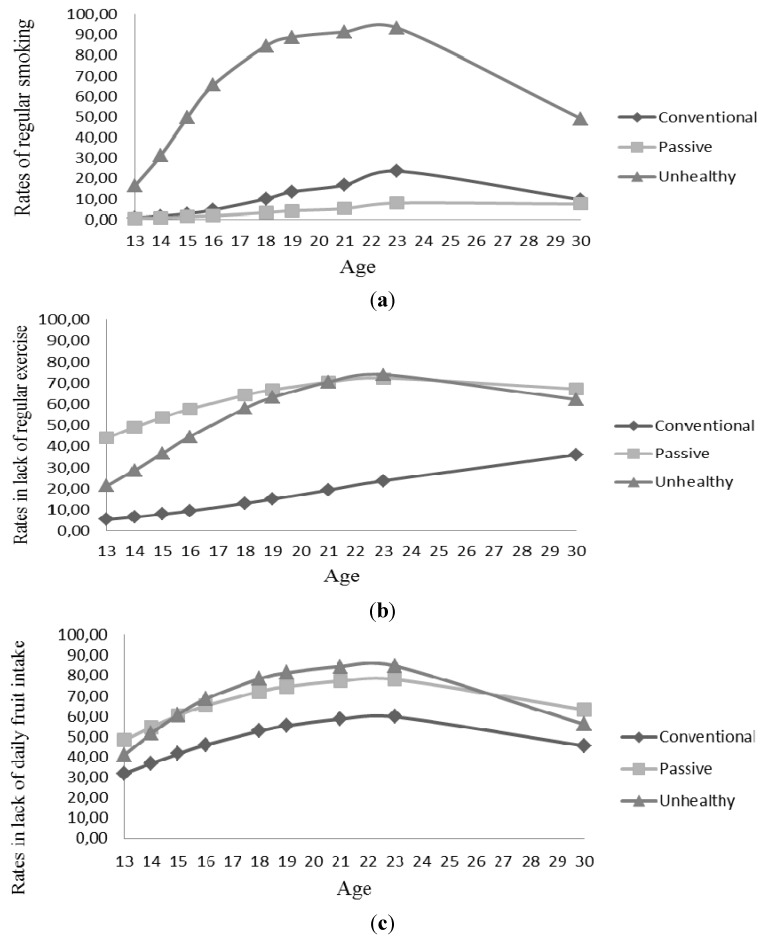

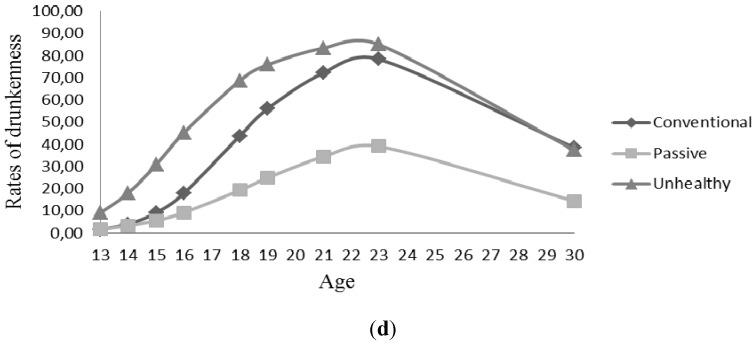
(**a**) Latent Class Growth Analyses of regular smoking (daily and weekly), (**b**) lack of regular physical exercise (physical activity once a week or less), (**c**) lack of daily fruit intake (six or fewer times a week) and (**d**) often drunk (at least five times drunk in the past six months), respectively: prevalence across a 17-year period (13–30 years).

We labelled the first trajectory (represented by the diamond marker) as the conventional trajectory due to a rather high degree of drunkenness during late adolescence/early adulthood coupled with reasonably healthy habits on the other health behaviors (Figure 1a–d). For drunkenness, the proportion of the conventional trajectory that reported getting drunk five or more times in the past six months increased throughout adolescence to about 78% at age 23 (Figure 1d), but then declined rather steeply to 38.5% at age 30. In contrast, the probability of smoking for the conventional trajectory was low throughout the entire period where the highest smoking prevalence (23.7%) was registered at age 23 (Figure 1a). Like regular smoking, low levels of regular exercise were registered (the lowest among the three trajectories) but lack of regular exercise increased over time and reached peak level of 36% at age 30 (Figure 1b). In Figure 1c, lack of daily fruit intake increased gradually until age 23 where about 60% were not eating fruit on a daily basis.

Using the third trajectory as the reference group, individuals in the conventional trajectory were more likely to have a father with higher educational background, a mother with lower educational background and more likely to be males (see Table 4); a closer inspection of the trajectories revealed that while sex compositions in trajectories 2 and 3 were comparable, there were clearly more males than females in the first trajectory (63% *vs.* 37%). There were no significant associations with the other covariates.

The second trajectory (represented by the square marker) was labelled the passive trajectory because it was associated with a rather low probability of engaging in both positive and negative health behaviors over time. The probability of smoking regularly was particularly low, with the highest smoking rate (8%) registered at age 23 (Figure 1a). Rather low levels of drunkenness (the lowest among the three trajectories) were also recorded with the highest level (39%) registered at age 23 (Figure 1d). However, and more comparable to trajectory 3, moderate to high levels of lack of regular exercise were registered with the highest level (72%) recorded at age 23 (Figure 1b). Similarly, high levels of lack of daily fruit intake, as found in trajectory 3, were found in this passive trajectory with peak level of 78% at age 23 (Figure 1c). No significant differences were found between trajectory 2 and the other trajectories regarding associations with the covariates that were studied (see Table 4).

The third or final trajectory (represented by the triangle marker) was described as the unhealthy trajectory because individuals following this trajectory had high and increasing levels of all four unhealthy behaviors. The probability of smoking was particularly high. Compared to the other trajectories, individuals following this unhealthy trajectory started smoking early and registered increasing levels of regular smoking (much higher than found for the other trajectories) with peak level of about 93% at age 23 (Figure 1a). Lack of regular exercise also increased and the highest rate (74%) was recorded at age 23 (Figure 1b). Moderate to high rates of lack of daily fruit intake (the highest among the three trajectories) were registered between late adolescence and the early adulthood, with the highest rate (85%) recorded at age 23 (Figure 1c). Like lack of regular exercise, rates in drunkenness, which were also the highest among the three trajectories, increased and reached peak level of 85% at age 23 (Figure 1d). Females, as well as individuals who had fathers with a lower educational level or mothers with a higher educational level were more likely to follow this third trajectory compared to the conventional trajectory. No significant associations with covariates were found when compared to individuals in the passive trajectory (see Table 4). 

**Table 4:** Associations between trajectories of unhealthy behaviors and covariates: multinomial logistic regressions.

Covariates	Trajectory 3 (Unhealthy) as Reference ^a^				Trajectory 2 (Passive) as Reference ^b^			
	*N* = 1057				*N* = 1057			
	Trajectory 1 (Conventional)		Trajectory 2 (Passive)		Trajectory 1 (Conventional)		Trajectory 3 (Unhealthy)	
	Estimate	Two-Tailed *p*-Value	Estimate	Two-Tailed *p*-Value	Estimate	Two-Tailed *p*-Value	Estimate	Two-Tailed *p*-Value
Sex	−0.715	0.038	−0.360	0.379	−0.355	0.407	0.360	0.379
Mother’s education	−0.302	0.035	−0.250	0.144	−0.052	0.763	0.250	0.144
Father’s education	0.300	0.030	0.218	0.191	0.081	0.631	−0.218	0.191
Have a child (children)	−0.053	0.885	−0.281	0.532	0.229	0.634	0.281	0.532
Married/ cohabiting	0.074	0.862	−0.412	0.404	0.485	0.351	0.412	0.404
Student (at 30)	−0.456	0.486	0.177	0.796	−0.632	0.408	−0.177	0.796

**Notes:** Loglikelihood: −13169.433; Sex: (1) male, (2) female; Have a child (children): (1) Yes, (2) No; Married/cohabiting: (1) No, (2) Yes; Student: (1) No, (2) Yes; **^a^** Trajectory 3 has the same estimates as trajectory 1 when the latter is treated as the reference group except that estimates have a positive sign instead of a negative sign and vice versa; **^b^** Trajectory 2 has the same estimates as trajectory 1 when the latter is treated as the reference group except that estimates have a positive sign instead of a negative sign and vice versa.

## 4. Discussion

Three growth trajectories of health behaviors from early adolescence to adulthood were identified in latent class growth analysis (LCGA): conventional, passive and unhealthy. The conventional trajectory appeared to reflect changes in physical activity, fruit intake, smoking and drunkenness according to the prevailing age specific norms of these behaviors in the Norwegian society at the time. The passive trajectory was characterized by individuals who reported low levels of both healthy and unhealthy behaviors during the 17-year period. Finally, the unhealthy trajectory indicated a pattern of high levels of unhealthy behaviors over time. Not all covariates were found to be significantly associated with the identified trajectories.

The conventional trajectory was characterized by increasing and high levels of drunkenness and increasing but low levels of regular smoking during late adolescence and early adulthood, while the rates of lack of regular exercise and lack of daily fruit intake during the entire study period were lower than in the two other trajectories. Thus, the individuals in this trajectory remained physically active and were likely to eat fruits daily throughout the period. According to Donovan *et al.* [38] greater involvement in health-maintaining behaviors, such as physical activity and fruit intake, has been shown to be related to greater conventionality. Experimenting with adult behaviors such as smoking tobacco (without establishing smoking as a habit) and drinking alcohol may also be regarded as conventional behaviors in late adolescence. This particular trajectory may be interpreted as conventional, as the individuals following this trajectory appeared largely to display a normative development of health behaviors than the two other trajectories, and may somewhat exemplify the trajectory of healthy behaviors suggested in the introduction. 

Consistent with our expectations, high rates of drunkenness during the transition from adolescence to young adulthood was observed to some extent, but the increase in drunkenness was found in all three trajectories, albeit in varying degrees. In the unhealthy trajectory, drunkenness and the other behaviors followed the same course of development. The high level of smoking is perhaps the most distinctive feature of this trajectory. Individuals in this trajectory reported taking up smoking at a very young age, with more than 50% reporting that they smoked at age 15. 

The passive trajectory was not hypothesized on the basis of previous theory and research. Individuals associated with this trajectory had a relatively low probability of engaging in both negative and positive health behaviors throughout the study period. Similar to individuals following the unhealthy trajectory, they were rather unlikely to exercise regularly throughout adolescence, and to eat fruit every day. Interestingly, their unhealthy lifestyle did not generalize to substance use; individuals following this trajectory were very unlikely to smoke regularly throughout the 17-year period, and even if the likelihood of being frequently drunk increased somewhat during late adolescence, the increase was less pronounced than for individuals following the other trajectories. 

### 4.1. Comparing Trajectories

The three trajectories share some common characteristics, in particular regarding drunkenness, which increased from age 13, peaks at age 23, and then decreased until age 30. This course of development has been identified in numerous studies during the past decades [23,24,26,39], suggesting a remarkable historical stability in rates of frequent heavy drinking in the transition to young adulthood. The sensation-seeking aspect of Steinberg’s [18] socio-emotional system may account for this trend. Schulenberg and Maggs [39] further suggest that alcohol use is inextricably linked to social relationships with peers. During late adolescence and early adulthood, many social activities occur in drinking contexts, and these interactions may be facilitated by alcohol. Sociability expressed while drinking can serve as a marker of successful peer relationships and social group bonding, and can be regarded as a normative behavior among individuals in their early twenties. However, for the majority, drunkenness subsides as they move into adult roles related to parenthood and paid work. 

The findings also suggest a negative development of physical activity and daily intake of fruits in the transition to young adulthood, perhaps reflecting increasing independence from their parents. Based on other analysis of data from the same study, Birkeland *et al.* [40] found that the majority leaves home at the age of 18-19 (at the end of secondary high school in Norway), and at the age of 23, 92% of women and 79% of men had left home. Thus, they are subjected to less parental influences on day-to-day living arrangements such as meals and physical activity, and are free to change into more health compromising habits. However, after age 23, daily fruit intake increased and physical activity rates stopped declining (with the exception of the conventional trajectory, but individuals in this trajectory were far more likely to be physically active than those in the other two trajectories during this age period); suggesting that, similar to the development of drunkenness, the majority mature out of unhealthy behaviors as they enter into adult roles. Parenthood has been claimed to be the main driving force behind this positive change [39], and more than 50% of the individuals in the present study indicated that they were parents at the age of 30. 

There were several differences as well between the trajectories. The unhealthy trajectory had higher levels of unhealthy behaviors throughout the study period compared to the other trajectories. Rates of lack of regular exercise and daily fruit intake were the lowest in the conventional trajectory, while the rates of drunkenness and smoking were the lowest in the passive trajectory. Moreover, individuals in the unhealthy trajectory were likely to start smoking and to get drunk at a younger age, and the increase in these behaviors was more pronounced than in the other trajectories. The clustering of unhealthy behaviors in the unhealthy trajectory supports previous findings on the co-occurrence of such behaviors [13,16,17]. Thus, individuals in this trajectory appear to be more at risk in terms of health outcomes, and therefore constitute important targets for health promoting interventions. 

Clustering of unhealthy behaviors was not observed to the same degree in the other trajectories, but appeared to be common only in a short period during late adolescence and early adulthood, and less so in the passive trajectory. Such trends as observed in the passive trajectory is seemingly not identified in previous studies, and could lead to increased insights into new and alarming trends in the development of health behaviors. It is possible that individuals in this trajectory are also passive in other contexts of life, such as in socializing with peers, civic participation, and school/work activities. Further research is required to replicate the existence of such a trajectory, and include other types of behaviors associated with a positive or negative development. 

### 4.2. Demographic Factors 

Cockerham [10] suggests that the interaction between life chances and choices will make individuals of high SES more likely to engage in health enhancing behaviors. In our findings, high educational level of fathers (as an SES marker) was associated with a trajectory that was characterized by a combination of low and high rates of unhealthy behaviors. A similar trend was found for high educational level of mothers although the combinations of unhealthy behaviors differed. Fathers’ and mothers’ educational level thus influenced engagement in health behaviors in different ways, although when considered together, parents’ high educational background generally tends to promote healthy behaviors [41]. Besides, the finding on females being more likely to follow the unhealthy trajectory may reflect earlier findings where early puberty in females was associated with risk behaviors such as substance abuse [42]. However, this association was not examined in the present study. Parenthood was expected to be associated with a trajectory of both unhealthy and healthy behaviors during the transition to adulthood, while not having children was expected to relate to a trajectory of increased drunkenness and unhealthy behaviors. The findings did not confirm these expectations, as parenthood status was not significantly associated with any of the trajectories. As all trajectories in general suggested a decrease in unhealthy behaviors after age 23, it is likely that parenthood is related to all trajectories, and thus not able to distinguish between them. Moreover, decline in physical activity or smoking, for example, have not only been found among parents or individuals becoming parents but also among late adolescents and the adult population in general [43]. The findings may also be due to the fact that we were not only examining the association between covariates and the developmental trajectory of one health behavior, but rather a trajectory of the health behavior together with three others. 

### 4.3. Limitations

Several limitations of the present study deserve mention. Self-report measures of health behaviors, such as those used in the present study may undermine the validity of the measures. This is because such self-report measures are usually affected by cognitive and situational factors [44]. However, self-report measures of health behaviors have also been found to adequately describe an individual’s perception of the behavior [45]. 

The comparable measures of the health behaviors that were used across the 17-year period may be considered a strength of the present study, except that the amount and intensity that are usually used to define the recommendation for some health behaviors were not specified. For example, what was implicitly considered as being physically active (*i.e.*, physical activity two or more times per week) may not be comparable to the World Health Organisation’s recommendation of at least one hour a day of physical activity for children and adolescents and 30 min for adults [46]. In addition, what was being referred to as lack of regular physical exercise comprised those who did not engage in physical activity and those who had lower levels of the behavior. Future studies may need a more stringent definition of physical activity and perhaps drunkenness and smoking. 

In growth mixture modelling, membership of classes is inferred from the data through a series of exploratory analyses undertaken to determine the best fit model [35]. The validity of the findings from such exploratory analysis may therefore be questioned [47] although the fit indices and theory that guide the analysis [48,49] may strengthen the legitimacy of the findings of such growth analysis. Moreover, it is important to emphasize that the existence of multiple classes in mixture modelling might be more due to skewed or non-normal data than the occurrence of qualitatively different populations [47]. It is also important to emphasize that the mixture models analyzed in the present study were highly complex and resulted in a heavy computational burden. Given both the explorative nature of the present analyses and the potential limitations mentioned above, it is important that the results from the present study are replicated in future studies. 

### 4.4. Implications for Intervention

Despite the above mentioned limitations, the identification of the different growth trajectories represent vital information that can be used in developing intervention strategies tailored to the needs of specific groups. From the present findings, individuals in the conventional trajectory appear to adhere to recommendations for physical activity and fruit intake, and very few establish smoking as a habit. Therefore, this may be considered a healthy group, perhaps less in need for interventions compared to the passive and unhealthy trajectories. However, the relatively high increase in drunkenness associated with the conventional trajectory represents a challenge for health promotion. At a younger age, the goal could be to delay the onset of normative adult behaviors, while at a later stage, efforts may be undertaken to diminish the potentially negative consequences of drunkenness.

For the unhealthy trajectory in particular, co-occurrence was identified among unhealthy behaviors. Females more than males, were more likely to follow this trajectory. Early puberty is one of several factors that have been found to be associated with unhealthy behaviors among females but this factor was not examined in the present study. To target risk behaviors co-occurring with early puberty in intervention programmes, future trajectory studies such as the present one would need to explore the effect of the onset of puberty and its association with engagement in unhealthy behaviors. Furthermore, intervention programmes that target multiple behaviors have been proposed. These intervention programmes may have a greater impact on public health compared to programmes that target single behaviors [50]. Because chronic diseases are usually a reflection of multiple unhealthy behaviors, designing interventions that will target multiple health behaviors may not only be cost effective but also the way forward.

## 5. Conclusions

While previous studies have generally focused on the course of development in individual behaviors such as physical activity or smoking, the present study contributes new insights into simultaneous patterning of the development of four health behaviors over a 17-year period. Application of an advanced statistical approach (the latent class growth analyses) made it possible to analyze growth trajectories of regular smoking, lack of regular exercise, lack of daily fruit intake, and drunkenness from early adolescence to adulthood, and to identify three distinct trajectories. The passive trajectory identified in this study appears particularly interesting and deserves further attention. It would be interesting to examine whether this developmental pattern of passivity in behaviors extends to other health behaviors, antisocial behaviors, as well as civic participation and participation in leisure activities. Trajectories similar to the conventional and the unhealthy trajectory in particular, have been identified in previous research on antisocial and problem behaviors. The present findings indicate that health enhancing and health compromising behaviors may also enter into such trajectories, reflecting age-limited processes. There are limitations however, that are related to the measurement of the health behaviors and the applied complex analysis, which can be addressed through more stringent measures of the behaviors and replications of the results in future studies.

Sex and parent’s educational level were found to be significant covariates that were associated with the different trajectories. The mechanism under which these factors operate together with others to promote or hinder health behaviors in young people is worth assessing in future studies. Other socio-demographic conditions such as civil status, having children, and occupational status were not found to be consistently related to the trajectories, suggesting the need for replication of the three trajectories. Future studies could include other psychosocial factors, such as relationship to parents and peers, school adjustment, and work experience, which may differentiate between the trajectories in a better way than the demographic factors included in this study. The different trajectories identified in the present study together with the co-occurrence of the health behaviors suggest the need for a health policy that takes into consideration tailored interventions, but also interventions that target multiple health behaviors.
